# Effectiveness of Intensive Linguistic Intervention in Autism Spectrum Disorder: A Case Study

**DOI:** 10.3390/children12020182

**Published:** 2025-01-31

**Authors:** Esther Moraleda-Sepulveda, Noelia Pulido-García, Nadia Loro-Vicente, Noelia Santos-Muriel

**Affiliations:** Faculty of Psychology, University Complutense of Madrid Campus de Somosaguas, 28223 Pozuelo de Alarcón, Spain; noepulid@ucm.es (N.P.-G.); nadialor@ucm.es (N.L.-V.); nosant02@ucm.es (N.S.-M.)

**Keywords:** Autism Spectrum Disorder, intervention, communication, language, case study

## Abstract

**Background:** Autism Spectrum Disorder (ASD) is currently classified as a neurodevelopmental disorder with increasing prevalence year by year. One of the key characteristics of this population is the persistent and variable difficulty they present in the development of functional language. For this reason, most individuals with ASD are candidates for linguistic treatment, especially during the early stages of development. The aim of this study was to assess the effectiveness of an individualized and intensive oral language and communication intervention. **Method:** This research was conducted through a case study of a 5-year-old Spanish-speaking child diagnosed with ASD. The child’s family sought intensive speech therapy to address articulation difficulties that were affecting speech intelligibility. However, a linguistic intervention program was proposed that would cover work in all areas of language. A comprehensive assessment of the child’s language and communication skills was carried out by a team of five professionals. Following this, an individualized intervention was implemented for 27 h per week over a period of 4 weeks. After this period, the child’s linguistic skills were reassessed. **Results:** The results show that the proposed intervention not only improved articulation skills. **Conclusion:** It is important to conduct a detailed analysis of each case and design individualized interventions that directly impact the effectiveness of treatments.

## 1. Introduction

Autism Spectrum Disorder (ASD) is a neurodevelopmental disorder that manifests in a variable range of alterations, primarily in social interaction, which in turn affects communication and language [1,2,3]. It develops at an early age, and failure to diagnose it early can impact an individual’s life for the long term [4]. Historically, ASD has been considered a disorder predominantly affecting the male population [5], with a prevalence four times higher in males than in females [3,6]. However, current studies suggest that this may be due to underdiagnosis in females [7].

Regardless of gender, it can be stated that individuals with ASD present communication difficulties, and some authors argue that a significant percentage of them never develop functional language [8,9,10]. A great deal of heterogeneity has also been documented regarding linguistic and communicative abilities in individuals with ASD, resulting in a spectrum ranging from the complete absence of conventional communicative behaviors to the use of complex structures, both in form and function [11,12,13]. Additionally, language difficulties appear at early ages and can affect all components of language [14].

Regarding the preverbal stage of language, it has been suggested that children with ASD are capable of requesting objects, actions, and social routines, persisting until they achieve their goal [15]. However, they seem to perform these communicative functions through protoimperative gestures, such as offering, showing, or pointing [16]. This has led some authors to suggest a dissociation between protoimperative and protodeclarative gestures [17], considering it an early sign of impaired social communication in children with ASD [18]. On the other hand, some authors have hypothesized that individuals with ASD show alterations in perceiving communicative acts, which could explain difficulties in responding to their own name [19,20]. Miller et al. [21] found that around 52% of children with ASD did not respond to their name at 12 months. It has also been documented that some individuals with ASD lack eye contact or make it in an atypical manner, leading to deficits in establishing joint attention later on and making the use of gestures as a primary form of communication difficult [22,23,24].

In relation to the phonetic–phonological area of language development, the speech of children with ASD is characterized by poor vocalization, which makes it difficult to understand, especially for individuals unfamiliar with their way of speaking [25,26,27]. In this regard, authors such as Cleland et al. [28] and Shriberg et al. [29] reported that approximately 41% of verbal children with ASD present phonological simplification processes, such as the absence of fricative consonants, affricates, velars, and liquids, and distortion or inconsistent use of nasals and alveolars [30].

In terms of morphosyntactic components, it is important to highlight the difficulties individuals with ASD face in understanding and using object pronouns, relative pronouns, and reflexive pronouns [31,32,33]. Moreover, their speech often includes prohibitive phrases, with negative or defiant statements [34]. The lack of logical sentence structure is another characteristic of their language, leading to a greater predominance of the present tense and difficulty in using and understanding verb tenses [35,36]. However, perhaps the most distinctive morphological feature is the use of pronominal inversion, understood as the use of the second or third person singular instead of the first, and using one’s own name to refer to oneself [37,38,39].

Regarding the semantic aspect of language, individuals with ASD are characterized by a discrepancy in expressive and receptive vocabulary scores, with expressive vocabulary scores being higher than receptive vocabulary scores [40,41]. Terms directed at social and emotional use are scarce, and they produce few statements aimed at conversation [42]. In addition to deficits in standardized vocabulary scores, children with ASD present unique deficits in specific subdomains of vocabulary, including the production and comprehension of personal pronouns, mental state terms, and prepositions [43]. They also face difficulties in understanding polysemy, as they struggle to understand why a word has multiple meanings [34].

Pragmatic language is undoubtedly the most altered aspect in individuals with ASD [44]. Among the most documented difficulties, it is important to highlight the use of echolalia and the limited creative language [45,46,47]. Echolalia refers to the repetition of decontextualized utterances made by individuals with ASD [44], which serve various communicative functions and regulate the nervous system [44,46]. Other authors have also reported difficulties in understanding indirect speech acts, metaphors, and jokes [48,49,50]. Additionally, they tend to ask repetitive or inappropriate questions [51,52,53], as well as engage in monolithic discourse and storytelling due to the cognitive inflexibility characteristic of this population [54].

In summary, it is important to consider not only the direct communication of individuals with ASD, but also ensure that different interlocutors are able to communicate with them and respond to their communicative intentions [10,55,56]. Therefore, communication and language should be approached as natural processes through more conscious interactions, promoting these processes as fundamental aspects for the development and learning of individuals with ASD [57,58,59]. For this reason, the objective of this work is to evaluate the effectiveness of individualized and intensive linguistic intervention in a child with ASD, taking into account their needs, interests and particularities and taking into account all areas of language and communication together [60,61,62].

## 2. Materials and Methods

### 2.1. Case Description

X.G. is a 5-year-old male, born in 2019, previously diagnosed with Autism Spectrum Disorder (ASD). He sought speech therapy in July 2024 for intensive therapy to improve his linguistic skills, particularly in the phonetic–phonological area and speech intelligibility.

An initial interview with the parents was conducted, during which they provided all existing reports to date. There were no abnormalities observed during the pregnancy. The pregnancy was full-term, and delivery was via cesarean section due to the baby’s position. He was breastfed until 8 months.

Motor development progressed normally. He sat up at 6 months, began crawling at 9 months, and walked independently at 12 months. His parents were concerned that he sometimes ran while looking sideways and on tiptoe. Additionally, the school recommended fine motor skill reinforcement.

Regarding language development, he began producing his first words after 24 months and started using 2- to 3-word phrases after 36 months, with speech characterized mainly by omissions and articulatory substitutions.

In terms of feeding, he is a selective eater and has stopped eating foods such as fruits and vegetables. Sleep-wise, he does not show alterations and sleeps well, taking a nap. By age three, he still did not show sphincter control.

X.G. started preschool at 13 months. During the first year, his socialization with other children was minimal. He was described as a very restless child, with difficulties staying seated and paying attention. This evolved into more regulated behavior, allowing him to follow routines in the classroom. He enjoys games like puzzles, playing with cars, and modeling clay.

In 2022, the family sought a psychological evaluation due to concerns about a possible neurodevelopmental disorder, referred by the pediatric service. The pediatrician noted delays in language development, inconsistent eye contact, limited response to his name, difficulty following instructions, frequent walking on tiptoe, and concerns about frequent touching of his neck.

The psychological evaluation was conducted using the Autism Diagnostic Observation Schedule-2 [62], the Bayley Scales of Infant and Toddler Development-3rd Edition [63], the Adaptive Behavior Assessment System-3 [64], and observation in natural settings, such as the school.

Results from the Bayley-3 Scales and the ABAS-3 revealed below-average cognition (16th percentile), linguistic ability well below expectations for his age (2nd percentile), impaired motor skills (12th percentile), and low general adaptive functioning (14th percentile), particularly in social (34th percentile), practical (21st percentile), and conceptual (2nd percentile) areas. The ADOS-2 scored a total raw score of 16 points, indicating symptoms consistent with Autism Spectrum Disorder, including communication difficulties, social interaction impairments, and restricted, repetitive behaviors and interests. Observations in the school context confirmed low frustration tolerance, motor stereotypies, and lack of social intent, with a strong tendency to play alone.

This information led to the diagnosis of Autism Spectrum Disorder. After the diagnosis, and due to the presence of linguistic and communicative difficulties, X.G. began attending speech therapy twice a week for 45-min sessions. Between January 2022 and June 2024, X.G. showed improvement in communicative intent, incorporating functions such as requesting, rejecting, greeting, and saying goodbye, although not consistently. His vocabulary and Mean Length of Utterance (MLU) increased, and he was able to imitate with gestural prompts and follow simple instructions. However, by April 2023, X.G. began to show more significant speech difficulties, marked by decreased intelligibility due to difficulties articulating phonemes both in repetition and spontaneously, as well as irregular voice volume.

Following the onset of these difficulties, X.G. was referred to the audiology service, where he underwent tympanometry, otoacoustic emissions testing, and auditory brainstem response (ABR) testing. Results revealed poor middle ear function bilaterally and altered cochlear mechanisms bilaterally. These findings were consistent with moderate bilateral hearing loss, although hearing aids were not prescribed.

Additionally, in April 2023, X.G. began supplementing traditional speech therapy with Applied Behavior Analysis (ABA) therapy, administered by a Certified Behavior Analyst once a week for 50-min sessions, focusing on social skills such as self-regulation strategies, personal space respect strategies, and communication strategies.

Finally, in 2024, due to persistent feeding restrictions, X.G. attended Occupational Therapy, where the Sensory Profile-2 [65] was administered. The results indicated a low auditory threshold, with high thresholds for visual, kinetic, and tactile stimuli, accompanied by a tendency for passive self-regulation, reflecting a pattern of low registration.

With all this information, the parents requested a case study to implement intensive language therapy in July 2024.

### 2.2. Assessment Instruments

To carry out the pre- and post-intervention assessment processes, a total of five assessment tools were employed. All tests used in the evaluation are standardized and validated instruments for the Spanish language.

First, the Objective and Criterion Language Battery Screening Revised [66] was administered. This test can be used between ages 5 and 14. This battery evaluates four different components of language: morphology, syntax, semantics, and pragmatics. Its purpose is to identify the areas where the individual has the most difficulty at the linguistic level. Regarding the reliability of the BLOC, Spanish studies report that the KR-20 coefficient of the semantic module is 0.90; that of syntax is 0.98; that of morphology is 0.98; that of pragmatics is 0.97. The evaluation experience has demonstrated its usefulness in populations with special educational needs [67].

Next, given the previously mentioned auditory difficulties, the subtests of phonological discrimination and recognition from the Revised Phonetic–Phonological Evaluation [68] were administered. This test is aimed at any age range.

The evaluation of the phonetic–phonological component was conducted using the Childhood Speech Phonological Evaluation [69]. This tool is used to detect phonological development disorders in children aged between 3 years and 7 years and 11 months through a set of cards that assess articulation.

The Communication Matrix [70] was then used to assess the pragmatic component. This tool is designed to measure communicative skills in individuals of any age whose communication is in the early stages. It allows for a precise determination of how the individual communicates. The matrix provides a list of communicative functions that the individual either masters, is developing (emerging), or does not have in their repertoire, based on 7 communication levels. The Communication Matrix is appropriate for individuals of any age who are in the initial stages of communication. The Matrix adapts to any type of communicative behavior, including augmentative modalities and communication alternatives (augmentative and alternative communication, AAC) and communication presymbolic (such as gestures, facial expressions, looks and body movements). It is appropriate for people with any type or degree of disability, including severe disabilities or multiple, intellectual limitations and sensory or physical disabilities.

Finally, the Phonological Knowledge Assessment Test [71] was administered. This test aims to assess, through 6 activities, the level of phonological knowledge (both syllabic and phonemic) of preschool children, as well as those who have difficulties with the acquisition of literacy, regardless of their educational level [71].

### 2.3. Procedure

This study was initiated following the request of X.G.’s family for intensive and specific intervention related to their child’s language difficulties. The family contacted the principal investigator of the EITAL research group (Research Team on Language Disorders and Alterations), which is composed of a team of professionals and researchers whose main focus is research and intervention in the field of speech therapy, specifically in children.

Subsequently, a coordination process was carried out between the EITAL team members and the child’s immediate environment (family and therapists). Several meetings were held before, during, and after the intervention process. The evaluation and intervention took place in July 2024, with a total duration of four weeks, from July 1 to July 26. This was conducted by five licensed speech therapists, specialized in child language. It is important to note that prior to the intervention, the parents were provided with an informed consent form regarding the use and treatment of their child’s data and images, which was completed.

A single-case design with pre- and post-intervention assessment was employed. The initial evaluation took place during the first week of the process, where the aforementioned tests were administered to establish X.G.’s language development level and determine the intervention goals. Based on this evaluation, five basic areas of focus were identified: articulation, intelligibility, phonological awareness, syntactic and semantic skills, and pragmatics. Each of these areas was addressed by a different speech therapist. The average number of hours spent by each therapist was approximately 21.6 h. The total treatment duration amounted to 108 h of work, with a weekly schedule of 27 h (5 to 6 h per day).

At the end of the intervention, a report with the results obtained by the child was compiled and delivered to the parents.

### 2.4. Intervention

To carry out the intervention, a treatment plan was developed by the five speech therapists, operationalized into objectives (Table 1).

### 2.5. Clinical Intervention

The clinical intervention followed a methodology based on learning through play [72,73] and consisted of a total of 88 h. During these sessions, the speech therapists interacted one-on-one with X.G. As we mentioned previously, the work team considered that, after analyzing the previous information and the initial evaluation, five large areas should be the focus of intervention in this case. These areas are: articulation, intelligibility, phonological awareness, syntactic and semantic skills, and pragmatics.

The intervention on speech and language precursors, articulation, intelligibility, and phonological awareness included activities such as auditory discrimination, sound searching and identification, syllable division, articulation of isolated phonemes, phonemes in direct syllables, and phonemes in monosyllabic, bisyllabic, trisyllabic, and polysyllabic words (increasing order of difficulty), structuring and formulating sentences, and activities involving unfamiliar or low-frequency vocabulary for X.G. During these activities, the therapists used evidence-based strategies to achieve the specific objectives being worked on. Some of the strategies employed included auditory bombardment [74,75], modeling [76], gestural facilitators [77,78], visual facilitators [79], expansions [80], and extensions [81].

The pragmatic work focused on the consistent use of communicative functions for behavioral regulation (requests and rejections) and social interaction (greetings, farewells, thanking, apologizing, and requesting permission). The objectives were hierarchized according to [82] proposal, beginning with ensuring the communicative functions already present in X.G. (although not used consistently), followed by the incorporation of new communication modes, the increase in content to be communicated, and the complexification of formal aspects. Since X.G. was able to imitate and benefited from gestural support, the Total Communication—Signed Speech Program by B. Scheaffer [83,84] was chosen. Thus, the communication intervention consisted of using communication temptations (based on the indications proposed by the ACACIA test [85] and the child’s interests), as well as modeling [86], physical shaping of communication signs [83], structured waits [87], parallel speech [88,89], expansions [80], and extensions [81].

### 2.6. Intervention in Natural Settings

In addition to the clinic-based intervention, the therapy was complemented with intervention in natural settings such as supermarkets, parks, and cafés. A total of 20 h of intervention were aimed at working in the natural context. Prior to these outings, anticipation and planning strategies [90] were used to explain to X.G. where they were going and what they would do. In the sessions prior to the natural environment outings (conducted in the clinic), situations that might arise were anticipated, and possible actions were discussed (e.g., “What do we do if we are not understood when we speak?”) using social stories [91]. Once in the natural setting, X.G. was exposed to communication with external conversation partners, practicing previously worked-on conflict resolution and communication skills. The aim was to raise X.G.’s awareness of his communicative and articulatory difficulties, while providing him with strategies that fostered his independence despite his challenges. The communicative functions of requesting, rejecting, thanking, and apologizing were worked on during these sessions.

Some of these sessions (7 h) were also conducted with the family environment, including X.G.’s sister as a key conversation partner [92]. In these sessions, the Teach-Model-Coach-Review protocol [93] was employed, with the aim of enabling X.G.’s sister to provide the necessary support for him to communicate effectively, rather than speaking for him when he was not understood. This approach aimed to enhance his autonomy.

Regardless of whether the sessions took place in a natural or clinical setting, coordination among the professionals was daily, with feedback provided to the parents to adapt the objectives and activities to X.G.’s individual characteristics and needs. Additionally, weekly meetings of the team were held.

## 3. Results

The results obtained by X.G. before and after the intervention are presented below. As previously mentioned, since this was an intensive intervention program addressing different areas of language and communicative development, a thorough evaluation of X.G. was conducted to establish a baseline and allow for subsequent comparisons. The following are the results obtained by X.G. in each evaluated area.

Regarding phonological discrimination, the results indicated no improvement, as the correct answer proportion remained the same (Direct score = 11/14; 78.57%) before and after the intervention. Specifically, phonological discrimination errors were found in the pairs of phonemes /p/ and /b/, /p/ and /f/, and /ɽr/ both before and after the intervention.

On the other hand, the results did show a slight improvement in phonological recognition following the intervention. Specifically, X.G.’s score on this task increased by 9 points, rising from a raw score of 133 before the intervention to a raw score of 142 after the intervention. Additionally, the results showed that the identification of most phonemes remained stable after the intervention, except for the identification of plosive vs. fricative phonemes and front vs. posterior fricative phonemes, which showed a higher increase in the direct score (3 and 4 points, respectively).

- Articulation and intelligibility

Moving on to articulation, the results showed a notable improvement with a significant reduction in speech simplification processes performed by X.G. Specifically, the child went from producing 16 different speech simplification processes before the intervention to only 6 after it. Therefore, as shown in Table 2, X.G. achieved correct articulation of the phonemes /ʝ/, /s/, /t/, /d/, /g/, /ñ/, as well as appropriate articulation of codas, syllabic onsets, and diphthongs after the intervention.

- Syntactic and semantic skills

Moving on to language development, the results demonstrated a clear improvement in the syntactic component. X.G.’s direct score increased by 12 points after the intervention, moving from −3.9 Standard Deviations (SDs) below children of his same chronological age to −0.8 SD after the intervention. However, the remaining components stayed below the expected level for his chronological age (between −1.4 SD and −5 SD compared to the normative group), although they remained relatively stable when comparing post-intervention results with pre-intervention results, as shown in Table 3.

- Phonological awareness

Continuing with phonological awareness, the results showed another improvement for X.G., as his total score increased from 9/30 before the intervention to 20/30 after it. Furthermore, Table 4 illustrates how X.G. improved in both phonemic and syllabic awareness.

- Pragmatic

Finally, in the pragmatic area, the results obtained by X.G. showed once again an improvement after the intervention. Specifically, as shown in Table 5, X.G. overcame pre-intentional behavior after the intervention and began to develop or improve unconventional communication, conventional communication, concrete symbols for communicative purposes, abstract symbols for communicative purposes, and language use.

Thus, the results reflect the improvement experienced by X.G. after the intervention. As can be observed, X.G. now consistently uses a greater number of concrete and abstract symbols for communicative purposes, as well as an increased number of joint attention and social interaction communicative functions.

## 4. Discussion

The case study presented has fulfilled the main objective set at the beginning of the research. Once again, it has been demonstrated how personalized and individualized intervention in language skills, based on the child’s characteristics, offers multiple benefits in improving the abilities and skills of individuals with ASD [94,95,96], especially in the early years of life [97,98]. Furthermore, it is noteworthy how, in this case, intensive intervention proved to be beneficial for the child, as indicated by other studies [99,100]. Pérez & Pérez [101] argue that three key characteristics of an effective intervention for ASD should be: (a) early initiation, (b) a high level of structure, and (c) an intensive nature, as also described by other authors [102,103,104], and these were implemented in the intervention proposed in this study. In line with this, Ratazzi [105] assert that if a child with ASD receives early intensive intervention, they have a chance of changing the trajectory of their development [106,107], which appears to be demonstrated by the post-intervention results.

It seems important, furthermore, that interventions conducted by specialists be complemented by work within the family environment. A meta-analysis on the effectiveness of language intervention in ASD [108] proposes that three factors determine the success of the treatment. First, early intervention, which enables children with ASD to significantly improve their language. Second, interventions should include training and feedback for parents [109]. And third, interventions should be long-term, allowing for adaptive interventions that monitor progress and adjust treatment according to the child’s response, particularly for children who start with minimal verbal skills [110]. The first two aspects were addressed in this intervention, and the third is currently being worked on, with continued monitoring and follow-up of the case.

Although individuals with ASD exhibit tremendous individual variability in language, especially in the early years [111,112], it appears that, as in this clinical case, the phonetic–phonological area is altered to a greater or lesser degree [30] and phonological intensive intervention can improve speech intelligibility to a greater or lesser degree, as in this case. Thus, improving intelligibility is one of the major challenges and goals that professionals focus on [113,114,115,116], following the family’s requests in this case. However, it is important to note that the intervention proposed by the professionals considered it essential to work on those areas of language that presented the greatest difficulties, understanding communication work as a global process encompassing all its aspects [59,117].

Regarding the proposed language treatment, this intervention has highlighted the direct and directional relationship between expressive language skills. Until now, much of the reviewed studies have focused on intervention programs specifically aimed at the pragmatic subsystem [118,119,120,121,122,123], but less attention has been given to language as a whole and the interconnectivity between language systems. Although initially the parents’ demand was to specifically work on articulating all the phonemes and their generalization to spontaneous speech, the team considered it necessary to address all the language areas that were affected to varying degrees in order to improve and consolidate the proposed objectives. The improvements have not only been significant in the phonological level, but the other language subsystems also benefited from this intervention. This aligns with theoretical studies that establish the relationship between phonetics and grammar [124,125], morphology [126,127], and semantics [128]. Nonetheless, it is important to emphasize that there are still few studies focused on phonetic–phonological intervention in ASD, and, as in the case of [129], they are also single-case studies. Similarly, some research has focused on improving articulation [130].

Language interventions in natural settings are increasingly seen as a way to enhance social communication skills in familiar environments [131,132]. Under the umbrella of social communication skills lies the need to increase functional, spontaneous expressive language in young children with ASD [133]. Research has also shown that naturalistic interventions promote social development, as they typically involve interactive exchanges between the child and an adult or typically developing peer [134]. Additionally, these are “family-friendly” approaches that tend to increase both the quantity and quality of early learning experiences. Parents can easily implement these strategies in their natural environments and during ongoing activities [135]. This work also aims to move toward natural setting intervention models to promote social inclusion [85].

Finally, it is worth highlighting that communication skills positively influence the quality of life of individuals with ASD and their families throughout all stages of development [136,137,138]. Therefore, long-term linguistic intervention should continue to be emphasized [139].

Regarding the limitations of this research, as it is a single-case design, the results cannot be generalized. However, it is considered essential that interventions be tailored to the level, characteristics, and environment of the person with ASD so that the direct impact of the intervention can be observed, thereby enhancing the child’s linguistic capacities and skills. It would be essential to monitor the child over a longer period of intensive therapy (3 to 6 months) to ensure the automation of speech and language patterns. This period allows for a more realistic assessment of the effects of intensive treatment due to the unique characteristics of the child’s psychophysiological development.

In conclusion, it is important to once again emphasize the necessity of implementing and evaluating interventions conducted in clinical practice to demonstrate their efficacy and effectiveness to the scientific community. To achieve this, it is important to study clinical cases in depth and value the interventions that are proposed from an individual and personal point of view. This will allow them to be used in the future by other professionals. Language intervention will not only help improve the language and communication characteristics of individuals with ASD in the early stages, but will also contribute to improving their quality of life throughout their development.

## Figures and Tables

**Table 1:** Proposed intervention objectives.

**Objectives Related to Speech and Language Precursors**
1. Auditory discrimination
- Improve the patient’s ability to audibly discriminate phonemes, syllables, and pairs of words.
2. Communicative elements
- Enhance eye contact so that the child can appreciate and interpret images and gestures. - Incorporate joint attention patterns by working on alternating eye gaze between the child and an object. - Encourage reciprocal gaze patterns to promote secondary intersubjectivity. - Optimize the understanding of the cause–effect relationship and the means–end connection. - Objectives related to speech.
**Objectives Related to Speech**
- Promote the use of tonal inflections during speech, emphasizing the melody of different types of sentences. - Improve vocal dynamics during speech to facilitate the capture of the listener’s attention. - Adjust speech speed and rhythm. - Increase speech intelligibility.
**Objectives Related to Language**
1. Phonetic–phonological area
- Improve the voluntary control of articulatory movements to achieve automation. - Achieve correct articulation points and modes for all the consonantal phonemes in Spanish. - Promote the identification of phonemes, their grouping into syllables, and their combination into words. - Foster the correct articulation of altered phonemes in syllables (both direct and inverse positions), words (monosyllabic, bisyllabic, trisyllabic, and polysyllabic), and sentences, reducing the processes of speech simplification detected: ○ Deaffrication of the consonant /ch/. ○ Occlusion of fricative sounds such as /x/ (replacing with /k/), /f/ (replacing with /p/), and /s/ (replacing with /t/). ○ Posteriorization of coronal sounds like /t/ (replacing with /k/) and /d/ (omitting or replacing with distorted /g/). ○ Voicing of sounds (/b/, /d/, and /g/ tend to be replaced by /p/, /t/, or /k/, or omitted). ○ Semi-consonantization of liquids (/kiase/ instead of /klase/). ○ Conversion of the approximant /ñ/ into a liquid (/nino/ instead of /niño/). ○ Absence of the trilled /r/. ○ Distortion of the trilled /r/. ○ Omission of final consonants or codas. ○ Omission of syllabic onsets at the beginning of words in polysyllabic words. ○ Simplification of diphthongs. ○ Simplification of complex syllabic onsets (/futa/ instead of /fruta/). - Generalize correctly produced phonemes into spontaneous language.
2. Morphosyntactic area
- Promote an Age-Appropriate Mean Length of Utterance (MLU). - Encourage the production of all the words in a sentence. - Achieve the use and generalization of function words. - Promote the production of more complex sentences, correctly joining sentences together. - Learn morphological variations of verbs and nouns regarding tense, number, and person.
3. Lexical–semantic area
- Encourage the integration of vocabulary according to the phoneme being worked on at the time. - Increase the understanding of word meanings and their integration into discourse.
4. Pragmatic area
- Promote the consistent use of communicative functions for behavioral regulation (requesting objects, actions, and rejecting) and social interaction (requests for social routines, greetings and farewells, showing, calling, requesting permission, and thanking). - Learn to use oral language functionally and contextually, thereby acquiring new modes of communication. - Increase the content to be communicated, enhancing the quantity of stimuli and conversation partners with whom communicative functions for behavioral regulation and social interaction can be used. - Complexify the formal aspects of communication by using combinations of words. - Incorporate the use of joint attention functions. - Promote positioning in the adjacent pair to balance conversational participation. - Foster conversational priority so the patient can provide semantically related responses. - Incorporate the use of compensatory editing tasks to improve intelligibility.

**Table 2:** Comparison of speech simplification processes before and after the intervention.

Substitution Processes		Pre-Intervention	Post-Intervention
Deaffrication	/ʃ/	x	x
Occlusion	/ʝ/	x	
	/f/	x	x
	/s/	x	
Posteriorization	/t/	x	
	/d/	x	
Voicing	/b/	x	x
	/d/	x	
	/g/	x	
Semi-consonantization of liquids		x	
Conversion /ɲ// to liquid		x	
Lateralization of vibrants			x
Omission of trilled r		x	x
**Syllabic Structure Processes**			
Omission of codas		x	
Omission of syllabic onset		x	
Simplification of diphthongs		x	
Simplification of complex onset		x	x

**Table 3:** Comparison between language skills before and after the intervention.

	Pre-Intervention			Post-Intervention		
	DS	CS	TS	DS	CS	TS
Morphology	1	1	3	0	1	0
Syntax	2	1	11	14	19	42
Semantic	6	1	36	4	1	21
Pragmatic	4	1	18	3	1	18

Note: DS: Direct score; CS: Centil score; TS: Transformed score.

**Table 4:** Comparison of phonological awareness skills before and after the intervention.

Skill	Pre-Intervention	Post-Intervention
Identification	6/10	8/10
Addition	3/10	9/10
Omission	0/10	3/10
Syllabic Awareness	5/15	11/15
Phonemic Awareness	4/15	9/15

**Table 5:** Comparison between pragmatic skills before and after the intervention.

	Achieved	Dominated	In Progress	Not Used
Pre-intentional behavior pre	100%			
Pre-intentional behavior post	100%			
Intentional behavior pre	0%	25%	0%	75%
Intentional behavior post	100%	0%	0%	0%
Unconventional communication pre	0%	75%	0%	25%
Unconventional communication post	0%	87.5%	12.5%	0%
Conventional communication pre	0%	7%	21%	72%
Conventional communication post	0%	29%	43%	28%
Concrete symbols pre	0%	12%	23.5%	64.5%
Concrete symbols post	0%	23.5%	57.2%	19.3%
Abstract symbols pre	0%	23.5%	23.5%	53%
Abstract symbols post	0%	35.3%	47%	17.5%
Language pre	0%	0%	35.5%	64.5%
Language post	0%	0%	41.2%	68.8%

## Data Availability

Data are not available due to privacy restrictions on information obtained from the participant.
